# Compliance of Healthcare Workers with Hand Hygiene Practices in Neonatal and Pediatric Intensive Care Units: Overt Observation

**DOI:** 10.1155/2014/306478

**Published:** 2014-11-25

**Authors:** Ayşe Karaaslan, Eda Kepenekli Kadayifci, Serkan Atıcı, Uluhan Sili, Ahmet Soysal, Gülcan Çulha, Yasemin Pekru, Mustafa Bakır

**Affiliations:** ^1^Department of Pediatrics, Division of Pediatric Infectious Diseases, Marmara University Medical Faculty, Istanbul, Turkey; ^2^Department of Infectious Diseases, Marmara University Medical Faculty, Istanbul, Turkey; ^3^Infectious Control Nurses, Marmara University Medical Faculty, Istanbul, Turkey

## Abstract

*Background.* The objective of this study was to assess the compliance of hand hygiene (HH) of healthcare workers (HCWs) in the neonatal and pediatric intensive care unit in a tertiary university hospital in Istanbul. *Methods.* An observational study was conducted on the compliance of HH for the five World Health Organization (WHO) indications. HCWs were observed during routine patient care in day shift. The authors also measured the technique of HH through hand washing or hand hygiene with alcohol-based disinfectant. *Results.* A total of 704 HH opportunities were identified during the observation period. Overall compliance was 37.0% (261/704). Compliance differed by role: nurses (41.4%) and doctors (31.9%) [*P* = 0.02, OR: 1.504, CI 95%: 1.058–2.137]. HCWs were more likely to use soap and water (63.6%) compared to waterless-alcohol-based hand hygiene (36.3%) [*P* < 0.05]. *Conclusion.* Adherence to hand hygiene practice and use of alcohol-based disinfectant was found to be very low. Effective education programs that improve adherence to hand hygiene and use of disinfectants may be helpful to increase compliance.

## 1. Introduction

Hand hygiene (HH) is one of the most effective methods of infection control programs, but compliance is generally poor. Hand hygiene improvement interventions must include control of compliance, which is mostly conducted by direct observation. Adherence to hand hygiene recommendations is the most important means to prevent and control the spread of healthcare associated infections (HCAI); however, adherence to hand hygiene practices is poor worldwide [1–3].

Both WHO and CDC guidelines recommend HCWs wash their hands with soap and water when visibly dirty; on the other hand, alcohol-based hand hygiene is recommended for all other opportunities [4, 5]. Alcohol-containing hand disinfection (AHD) is an effective alternative to standard soap and water. The aim of this study is to investigate the compliance of HH among the HCWs in neonatal and pediatric intensive care units in a tertiary university hospital.

## 2. Methods

Observational HH data were collected from two wards including a pediatric intensive care unit and a neonatal intensive care unit, during a month, 1-hour observations period by a doctor from pediatric infectious disease department working with infection control team from June 2013 to July 2013. The observer was placed inside the patient's room. HH compliance of HCWs for “My Five Moments for Hand Hygiene” (MMH) of the World Health Organization (WHO), which define the key moments when healthcare workers should perform hand hygiene indications, was determined by observer [5]. The five moments identified in this strategy include (1) prior to patient contact, (2) prior to a clean or aseptic procedure, (3) after contact with body fluid, (4) after patient contact, and (5) after contact with the patient environment. Hand hygiene (HH) is one of the basic components of the infection control program. Hand washing sinks were situated both in the wards and in the entrance of the wards. Alcohol-based disinfectants were provided at each sink and one for every one bed or incubator and also available outside the patient rooms. The use of both waterless, alcohol-based hand disinfectants and hand washing with soap was accepted to have equal efficacy in our study; however, alcohol-based disinfectants provide a residual effect that soap and water do not provide. HH opportunities and attempts were designated as appropriate or inappropriate per WHO criteria.

## 3. Results

A total of  704 hand hygiene opportunities were collected from the neonatal and pediatric intensive care units in Istanbul, Marmara University, Pendik Training and Research Hospital, from June 2013 to July 2013. Overall HH compliance was 37.0% (261/704) in HCWs. Compliance differed by role was as follows: nurses, 41.4%, and doctors, 31.9% [*P* = 0.02]. Overall HH compliance with respect to the five MMH were as follows: overall compliance of prior to patient touching was 43.2%, prior to a clean/aseptic procedure was 8.5%, after body fluid exposure was 18.1%, after contact with patients was 68.1%, and after contact with patient surroundings was 43.2%. HCWs mostly prefer to wash their hands with soap and water procedure. HCWs were more likely to use soap and water (63.6%) compared to alcohol-based hand hygiene disinfectant (36.3%) [*P* < 0.05]. The compliance and the technique of hand hygiene are shown in the Table 1.

## 4. HH Compliance among Nurses

Overall compliance of HH in nurses was 41.4%. Nurses mostly prefer to wash their hands after touching a patient (74.6%) [61.1% by hand washing with soap and water and 13.4% by hand hygiene with disinfectant], secondly, after touching patient surroundings (60.1%) [45.5% by hand washing with soap and water and 14.6% by hand hygiene with disinfectant], thirdly, prior to touching the patient (43.8%) [21.0% by hand washing with soap and water and 22.8% of by hand hygiene with disinfectant], fourthly, after body fluid exposure risk (16.8%) [10.3% by hand washing with soap and water and 6.4% of hand hygiene with disinfectant], and lastly prior to a clean/aseptic procedure (7%) [2.3% by hand washing with soap and water and 4.7% by hand hygiene with disinfectant].

## 5. HH Compliance among Doctors

Overall compliance of HH in doctors was 31.9%. Doctors mostly prefer to wash their hands after touching a patient (55.5%) [27.7% by hand washing with soap and water and 27.7% of by hand hygiene with disinfectant], secondly, before touching patient (48.7%) [21.9% of by hand washing with soap and water and 26.8% of by hand hygiene with disinfectant], thirdly after touching patient surroundings (28.5%) [11.9% of by hand washing with soap and water and 16.6% of by hand hygiene with disinfectant], fourthly, after body fluid exposure risk (22.7%) [18.1% of by hand washing with soap and water and 4.5% of by hand hygiene with disinfectant], and lastly before a clean/aseptic procedure (12.5%) [6.2% by hand washing with soap and water and 6.2% of by hand hygiene with disinfectant].

## 6. Discussion

According to a wide point prevalence survey of the ECDC (European Center for Disease Control and Prevention), they reported that approximately 80,000 patients—or one in 18 patients—in European hospitals have at least one healthcare-associated infection. In Turkey there are limited data; according to previous studies the hospital prevalence of healthcare-associated infections was reported to be 13.4% [6], reaching 48.7% in intensive care units [7]. However, in 2009, the Ministry of Health of Turkey began a hand hygiene campaign, called “Danger in Your Hands,” throughout the country. The aim of this campaign is to improve the hand hygiene compliance in healthcare settings. Since 2006, all hospitals in Turkey report their hospital infection rates through surveillance system that is organized by the Refik Saydam Hygiene Center. Since that time, the hand hygiene campaign has been implemented in many hospitals. In our hospital, there is a hospital-wide hand hygiene promotion campaign that is repeated at regular intervals.

Patients in the intensive care units (ICUs) are more likely to be infected by multidrug-resistance microorganisms and most of these infections are spread by carriage of microorganisms on the healthcare workers' (HCWs) hands [8]; outbreaks of infections resulting from cross-transmission are frequent [9].

Hand hygiene, before and after all patient or patient environment contact, before aseptic procedure, and/or after body fluid exposure, which are WHO indications, is recommended in all published infection control and public health guidelines and is considered the standard of care for all HCWs [10–15]. On the other hand, compliance with hand washing is still poor [16, 17]. The present study aimed to determine the compliance with HH among health workers. There are many studies that document the compliance hand hygiene among HCWs. In Europe compliance to HH differs in the reports ranging from 33 to 65% [18–20]. In Turkey, there are few studies that evaluate the compliance of HH. In the study of Karabey et al., the frequency of hand washing was 12.9% among medical personnel in an intensive care unit [21]. Sacar et al. observed that hands were washed both before and after venipuncture in only 41 (45.1%) cases [22]. These compliance rates are very low similar to the data in the literature.

Similar to many studies in the literature, compliance with HH among nurses is better than doctors [23, 24]. The current study also reports similar results. Furthermore, the current study attempted to evaluate the use of hand hygiene techniques based on the WHO five indications. The researchers noticed that most of the HCWs prefer to use HH after patient contact or contact with the patients' environment, in contrast to a very low rate for prior to patient contact. These findings lead to the assumption that HCWs prefer to protect themselves rather than patients.

The WHO Guidelines on Hand Hygiene in Healthcare and the Centers for Disease Control and Prevention in the United States recommend direct observation of compliance and measuring the consumption of HH products [1, 11, 25]. Direct observation helps to pinpoint the areas of strength or weaknesses in HH behavior, to identify the number of HH opportunities and their indications, to assess techniques, and to provide feedback to healthcare workers (HCWs) [5, 26]. Similar to many studies in the literature, the researchers of the current study also prefer to measure the compliance of HH by direct observation. However, there is potential bias occurring from hand hygiene direct observations. One of the most important ones is the Hawthorne effect, which is attributed to the tendency of people being observed in a research context to behave differently from the way they would otherwise [27]. When the HCWs know that they are under observation, HH performance usually improves. However, in the present study, although HCWs knew that they were under observation, overall compliance of HH remained very low. Observer and selection bias can be minimized by validated observers, randomly choosing locations, HCWs and day shifts [5]. New technologies should be investigated and promotional educational programmes must be tailored to improve HH compliance.

HCWs were more likely to use soap and water compared to waterless alcohol-based hand hygiene in our study similar to some of data in the literature; however, alcohol-based disinfectants provides a residual effect that soap and water do not provide [18, 28]. We think that alcohol-based disinfectants were not preferred because of the unpleasant irritation effects on the hands and lack of knowledge concerning its benefits.

## 7. Conclusion

Although the HH procedure is simple, HH compliance among HCWs is so low that it cannot be easily explained or changed. The authors believe that a lack of motivation and increased workload may be the two causes of poor compliance. In the present study, the highest compliance rates were after patient contact and contact with the patient environment, and for this reason the authors believe that HCWs prefer to protect themselves to a greater extent than the patients.

## Figures and Tables

**Table 1:** Compliance and technique of hand hygiene among nurses and doctors.

	Total *n*, (%)		Nurses *n*, (%)		Doctors *n*, (%)		*P* value, OR (95% CI)
	Hand washing with soap and water	Hand hygiene with disinfectant	Hand washing with soap and water	Hand hygiene with disinfectant	Hand washing with soap and water	Hand hygiene with disinfectant	Hand washing with soap and water and with disinfectant
Prior to patient contact	33 (20.4%)	37 (22.8%)	24 (21.0%)	26 (22.8%)	9 (21.9%)	11 (26.8%)	0.719, 0.820 (0.401–1.678)

Prior to a clean or aseptic procedure	4 (3.4%)	6 (5.1%)	2 (2.3%)	4 (4.7%)	2 (6.2%)	2 (6.2%)	0.458, 0.532 (0.140–2.023)

After contact with body fluids	12 (12.1%)	6 (6.1%)	8 (10.3%)	5 (6.4%)	4 (18.1%)	1 (4.5%)	0.540, 0.691 (0.216–2.207)

After patient contact	46 (52.3%)	14 (15.9%)	41 (61.1%)	9 (13.4%)	5 (27.7%)	5 (27.7%)	0.199, 2.353 (0.799–6.931)

After contact with the patient environment	71 (29.8%)	32 (13.5%)	56 (45.5%)	18 (14.6%)	10 (11.9%)	14 (16.6%)	0.001, 3.776 (2.082–6.847)

Total hand hygiene compliance among nurses and doctors							0.02, 1.504 (1.058–2.137)
